# Impact of Cassava Starch Varieties on the Physiochemical Change during Enzymatic Hydrolysis

**DOI:** 10.3390/molecules27186098

**Published:** 2022-09-18

**Authors:** Fabiola Cornejo, Pedro Maldonado-Alvarado, Sócrates Palacios-Ponce, David Hugo, Cristina M. Rosell

**Affiliations:** 1 Escuela Superior Politécnica del Litoral, ESPOL, Facultad de Ingeniería en Mecánica y Ciencias de la Producción, Campus Gustavo Galindo, Guayaquil P.O. Box 09-01-5863, Ecuador; 2 Department of Food Science and Biotechnology, Escuela Politécnica Nacional, Quito P.O.·Box 17-01-2759, Ecuador; 3 Department of Food and Human Nutritional Sciences, University of Manitoba, Winnipeg, MB R3T 2N2, Canada; 4 Instituto de Agroquimica y Tecnologia de Alimentos (IATA-CSIC), 46980 Valencia, Spain

**Keywords:** enzymatic modification, hydrolysis degree, starch modification

## Abstract

The enzymatic modification of starch extends its industrial use to flavor delivery and probiotic encapsulants, among other uses. However, it is not known how starch from different cassava varieties responds to enzymatic hydrolysis. Starches from two Ecuadorian cassava varieties (INIAP 650, an edible starch, and INIAP 651, an industrial starch) were partially modified at three enzymatic hydrolysis degrees (0%, 30%, and 50%), and their physicochemical properties were assessed. The structural analysis revealed that both varieties showed progressive structural damage as hydrolysis increases, probably due to exo-hydrolysis. However, deeper pores were observed in INIAP 651 with the SEM analysis. The crystallinity percentage obtained by XRD analyses remained constant in INIAP 651 and decreased (by 26%) in INIAP 650 (*p* < 0.05). In addition, the amylose–lipid complex index in INIAP 650 remained constant, while INIAP 651 increased (*p* < 0.05) at 30% hydrolysis (by 93%). In both varieties, hydrolysis increased (*p* < 0.05) the water holding capacity (WHC) (by 10–14%) and the water binding capacity (WBC) (by 16%), but 50% hydrolysis of INIAP 650 was needed to significantly affect these properties. No differences were observed in the varieties’ thermal properties. Regarding the rheological properties, the variety did not influence the changes in the storage module (*G*′) and the loss modulus (*G*″) with the hydrolysis (*p* > 0.05). However, the phase angle decreased significantly (*p* < 0.05) with the hydrolysis, being higher in the INIAP 650 variety than in the INIAP 651 variety. In general, the results indicate that the variety affects the response of the starch granule to enzymatic hydrolysis (noticeable in the principal component analysis, PCA) and opens up the possibility to modulate starch properties.

## 1. Introduction

Starch is a natural polymer widely distributed in nature which can be used industrially in food and non-food products. The industrial use of starch requires physical, chemical, and enzymatic modification, or their combinations, to improve its technological properties, such as hydration, rheological, and thermal properties, among others.

The partial enzymatic hydrolysis of starch is a surface modification method, which allows obtaining porous starch. Several studies have been carried out to optimize enzymatic hydrolysis. Amylolytic enzymes, branching enzymes, and their combination have been used for starch hydrolysis [1,2,3]. Some authors state that the botanical source, the enzyme type, and its concentration impact porous corn starch’s structural and physicochemical properties [1,3,4].

In fact, the combination of the enzyme type, enzyme concentration, reaction time, and the degree of starch hydrolysis affects the dimension and type of porosity [5,6]. Guo et al. [2] showed that combining three enzymes increases porous starch’s absorption capacity. Some studies have revealed that the digestibility of porous starch is also affected by hydrolysis conditions [4,7,8]. In addition, Benavent-Gil and Rosell [4] compared the effect of enzymatic hydrolysis on different botanical sources, showing that porous starches from cereal and tubers presented different structural and functional characteristics, which also depended on the type of enzyme [4].

Cassava is a starchy root that can be used for culinary or industrial purposes depending on its variety, which defines the yield of cassava starch and its functional and physicochemical properties. The cassava variety influences the starch’s hydration, thermal, rheological, and pasting characteristics [9,10]. To the authors’ knowledge, no previous studies have been conducted to identify the impact of the starch variety at different hydrolysis degrees. Therefore, this paper aims to analyze the effect of cassava starch varieties on the structural and physicochemical properties at different degrees of enzymatic hydrolysis. Two Ecuadorian starch varieties with different applications (culinary or industrial purposes) were studied.

## 2. Materials and Methods

### 2.1. Cassava Starch and Enzymes

Two Ecuadorian cassava (*Manihot esculenta Crantz*) varieties, INIAP 650 and INIAP 651, created and obtained from the Ecuadorian National Institute of Agricultural Research (INIAP, Portoviejo, Ecuador), were used. Both varieties were harvested in May 2018. INIAP 650 is derived from the MCol 2215 clone, and INIAP 651 is derived from the CM 1335-4 clone [11]. The starches were characterized previously by Silva [11]; the author found significant differences between varieties [11]. The chemical composition of INIAP 650 was moisture 11.3%, protein 1.2%, fiber 4.3%, fat 1.9%, ash 1.4% and CHO 79.9% and for INIAP 651 was moisture 14.5%, protein 2.1%, fiber 6.0%, fat 1.5%, ash 1.3% and CHO 74.6% [11].

Starch extraction was done traditionally as is currently carried out in Manabí, Ecuador. The cassava roots were manually peeled and grated in a craftmanship grater. Then, the ground material was washed with a linen-type cloth. The filtrate was sedimented and the precipitate was collected and dried in a solar dryer at 29 °C for 24 h. The starch was stored at room temperature.

Commercial fungal enzymes were used for starch hydrolysis. Fungal enzymes, alpha-amylase (AM) from *Aspergillus oryzae* (Enzymix 5000), and glucoamylase (GA) from *Aspergillus niger* (Granozyme FGDX CAL) were supplied by GRANOTEC Company (Guayaquil, Ecuador). The enzyme activity units for AM and GA were 5000 SKB/g starch and 500 FAU/g starch, respectively.

### 2.2. Degree of Starch Hydrolysis

The in vitro starch hydrolysis was performed to establish the time at which 30% and 50% of hydrolysis degrees were reached, according to the method described by Jung et al. [12]. Starch slurry (10% *w*/*v*) in 0.1 M sodium acetate buffer (pH 4) was mixed with both AM and GA enzymes at a 1:3 ratio (AM: GA). The hydrolysis was carried out at 50 °C and 165 rpm for 24 h. A small aliquot was extracted from each experimental treatment at 0, 2, 4, 6, 8, 16, and 24 h of hydrolysis to measure reducing sugar expressed as free glucose, using the dinitrosalicylic acid (DNS) method. At each time, the hydrolysis was stopped by adding NaOH solution at 4% (*w*/*w*). Absorbance was measured at 540 nm using a microplate reader (Biotek Instruments, Winooski, VT, USA). All tests were performed in triplicate. The following equation calculated the starch hydrolysis degree:

Hydrolysis degree (%)=G1G2×f1×100

where *G*_1_ is the weight of total released reducing ends equivalent to free glucose (g) after hydrolysis, *G*_2_ is the weight of the raw starch sample (g) at the beginning of hydrolysis, and *f*_1_ is the conversion factor of free glucose to anhydroglucose, as occurs in starch equal to 0.9.

According to the hydrolysis kinetics, 30% hydrolysis was reached in 8 h (32.36 ± 1.58% in INIAP 650 and 32.70 ± 1.86% in INIAP 651) and 50% hydrolysis was reached at 16 h (50.95 ± 1.57% in INIAP 650 and 49.73 ± 2.01% in INIAP 651). After each hydrolysis process at 8 and 16 h, the dispersion was decanted for approximately 45 min at room temperature. Then, the modified starch was washed twice with deionized water and then dried in a convection oven at 50 °C for 20 h. Finally, the dried sample was ground and stored at room temperature. Thus, the applied treatments for analysis were 0%, 30%, and 50% of hydrolysis.

### 2.3. Structural Properties

The X-ray diffraction analysis was performed in an X’Pert PRO diffractometer (PANalytical, Boulder, CO, USA) at 30 mA and 40 kV. A 2θ diffraction angle from 5 to 40° with a 0.03 step size was applied. Before the analysis, starch was hydrated in a closed chamber at 100% relative humidity for 24 h. The relative crystallinity (RC) was calculated with the software Origin 2021 (OriginLab Corporation, Northampton, MA, USA) using the equation: RC (%) = (Ac/(Ac + Aa)) * 100 where Ac is the crystalline area and Aa is the amorphous area on the X-ray diffractograms.

The starch microstructure was observed in a scanning electron microscopy (SEM) (FEI, Inspect F, Hillsboro, OR, USA) coupled with energy dispersive X-ray spectrometry (EDS) at accelerating voltage of 10.5 kV with 1000×, 2500×, and 5000× magnifications. The samples were coated with gold. The particle size was measured using Image J 1.53c software (National Institutes of Health, Bethesda, MD, USA).

### 2.4. Physicochemical Properties

The amylose content was measured according to the method described by Hoover and Ratnayake [13]. The complexing index of the amylose–lipid complexes was measured by applying Tang and Copeland’s [14] method with a slight variation. A starch solution of 10% (*w*/*w*) was heated at 95 °C for 30 min at 160 rpm. The paste was mixed with 25 mL of distilled water. The solution was centrifugated at 4500 rpm for 15 min. An aliquot of the supernatant was mixed with distilled water and iodine solution. The absorbance was measured at 690 nm within 60 min after adding the iodine solution.

The starch hydration properties (water binding capacity, WBC; water holding capacity, WHC), and starch gelling behavior (water absorption index, WAI; water solubility index, WSI; and oil absorption capacity, OAC) were measured according to the methodology described in Cornejo and Rosell [15].

The thermal properties of the starch, such as onset temperature (To), peak temperature (Tp), and conclusion temperature (Tc), including enthalpy of gelatinization (ΔH), were calculated in a differential scanning calorimeter (Q-200, TA instruments, Newcastle, DE, USA). The modified starches were mixed with water at a ratio of 1:3 (*w*/*w*) and placed in an orbital shaker (Thermo Scientific, Waltham, MA, USA) at 150 rpm for 90 min. Then, the slurry (8 ± 0.1 mg) was placed in an aluminum hermetic pan. The pans were heated from 25 to 100 °C at a rate of 10 °C min^−1^.

The rheological properties of the starch gels were measured using a Kinexus PRO rheometer (Malvern Instruments, Worcestershire, UK). Starch slurry (0.5 ± 0.01 g in 4.5 mL of distilled water) was heated at 90 °C for 10 min. The storage modulus (*G*′), loss modulus (*G*″), and phase angle (tan δ) were measured at 25 °C with a cone-plate geometry (diameter of 4 mm; angle of 4°).

For statistical analysis, *G*_1_′ and *G*_1_″ were documented at a frequency of 1 Hz. The linear viscoelastic region was estimated at 0.1%. Three replicates were made for each sample.

### 2.5. Statistical Analysis

A factorial arrangement was developed considering two starch varieties and three different hydrolysis degrees. An analysis of variance (ANOVA) was used to determine significant differences with 95% confidence (*p* < 0.05) between studied factors. Fisher’s least significant differences (LSD) test was used to compare means in the parametric data, and the Kruskal–Wallis test was used to compare medians in the non-parametric data, with 95% confidence (*p* < 0.05) in physicochemical properties. The results are presented as mean ± standard deviation (SD).

Additionally, Pearson correlation coefficients analysis was performed among variables. Only coefficients greater than 0.68 were considered. A principal component analysis (PCA) was carried out to identify groups of samples with similar characteristics. All statistical analyses were performed using Statgraphics Centurion 16 (Statgraphics Technologies, Inc.; The Plains, VA, USA).

## 3. Results and Discussion

### 3.1. Structural Properties

**Figure 1:**
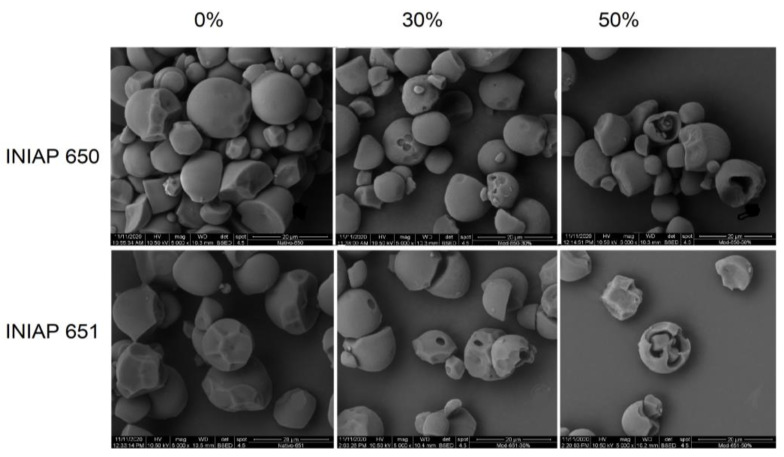
Scanning electron micrograph of two cassava starch varieties (INIAP-650 and INIAP-651) hydrolyzed at 0%, 30%, and 50%. Magnification 5000×.

Figure 1 shows the scanning electron micrographs for the cassava starch varieties (INIAP 650 and INIAP 651) hydrolyzed at 0%, 30%, and 50%. Native starches of both varieties displayed truncated granules and some grooves due to the compaction in the cassava. The enzymatic hydrolysis was evident in both starches, which displayed marked circled holes in the surface of the starch granules. In some granules, the shell granule remained during hydrolysis. Significant structural granule damage was evidenced at 50% of hydrolysis. Chen and Zhang [5] demonstrated that the degree of hydrolysis increases the pore size of corn starch. Likewise, Benavent-Gil and Rosell [1] showed that enzyme concentration influences pore size and shape, but these were also dependent on the enzyme type. Deeper pores were observed in the INIAP 651 variety. In both varieties, the starch granule surface remained smooth, which could be attributable to the hydrolysis being carried out at the surface level, i.e., exo-hydrolysis. Indeed, the starch particle sizes D (4, 3) of the INIAP 651 cassava variety decreased significantly as the hydrolysis degree increased (Table 1), but no significant effect was observed in those from INIAP 650 cassava variety. The variance analysis showed that only the degree of enzymatic hydrolysis influenced granule particle size reduction (*p* < 0.05).

The diffractogram of native and hydrolyzed cassava starch (data not shown) demonstrated that the cassava starches presented an A-type diffraction pattern with strong reflections at 2θ: ~17°, 26°, and an unresolved doublet at ~19° and 21°. No significant changes were observed in diffraction spectra and peak positions with different hydrolysis degrees and between varieties. Table 1 shows that the variety and degree of hydrolysis influenced starch crystallinity (*p* < 0.05). An increase in crystallinity was reported previously in porous starches, suggesting that hydrolysis occurs in the amorphous region of the starch [8,16,17]. Nevertheless, our results indicate that starch crystallinity in the INIAP 651 variety did not significantly vary during hydrolysis, while the INIAP 650 variety showed a significant reduction (*p* < 0.005) at 50% hydrolysis. Uthumporn et al. [18] reported a decrease in cassava starch crystallinity at 24 h of hydrolysis with a mix of AM and GA. They suggested that part of the amylopectin crystalline region could be degraded in cassava starch [18]. Recently, Prompiputtanapon et al. [19] reported a decrease in cassava starch’s relative crystallinity when AM and both AM, and AMG (amyloglucosidase) were used. They indicated that AM could partially penetrate the crystalline region, decreasing the relative crystallinity. They attributed results to the enzyme dosage, incubation time, and degree of hydrolysis. In addition, Benavent-Gil and Rosell [4] demonstrated that the botanical sources and the type of enzyme also influence the structural and functional features of porous starches. Our results indicate that the starch structure changed during hydrolysis in INIAP 650. Although the native starches of both varieties have the same degree of crystallinity, the crystalline zone in INIAP 650 is partially degraded during enzymatic hydrolysis. Therefore, variety influences the starch granule response to enzymatic hydrolysis.

### 3.2. Physicochemical Properties

Table 1 shows the hydration, thermal and rheological properties of the INIAP 650 and 651 cassava starches hydrolyzed at 0%, 30%, and 50%. Even though both varieties are derived from different clones, no significant differences (*p* > 0.05) in hydration properties and amylose content were observed in native starch. The results show a considerable increase of amylose content with hydrolysis (*p* < 0.05). The ANOVA analysis shows that the variety (*p* < 0.001) and the hydrolysis process (*p* < 0.001) influence the increase of amylose content. The increase of amylose content was more significant in the INIAP 651 variety (~25%) than in the INIAP 650 variety (~20%). Previous studies in corn, cassava, potato, wheat, and rice reported that enzymes such as AM, GA, AMG, and MA (maltogenic α-amylase) preferentially hydrolyzed amylose chains; thus, a decrease in apparent amylose content was expected [7,16,18]. Some authors have reported that an exo-type amylolytic enzyme such as GA rapidly hydrolyzes the amorphous region of corn starch, where amylose is mainly found [5,12]. Other studies in corn starch showed no change or reduction in amylose content when AM was used [1,4,8]. Furthermore, controversial findings have been observed when AMG is used in rice and corn starch; some authors reported increased and others reduced amylose content [1,4,16,20]. Uthumporn et al. [18] reported no significant change in amylose content when AM and GA were used simultaneously in cassava starch [18]. Divergences might be explained due to the different starch sources, enzymatic treatment, or even source varieties. The present study suggested higher action on the amylopectin content, which increased the apparent amylose content. In fact, no correlation was found between amylose content and crystallinity, which is associated with amylopectin. Some authors reported that GA could hydrolyze the terminal α 1-4 glucosidic linkage of glucose from the non-reducing ends of the starch granule [21,22]. Thus, the synergic effect of GA and AM in cassava starch might quickly attack the amylopectin non-reducing ends, decreasing amylopectin content and showing an apparent increase in amylose content. The exo-hydrolysis of GA could facilitate the endo-hydrolysis of AM in the α 1-4 glucosidic bond. In addition, longer amylopectin double helices can be formed when amylopectin branches are shorter [23]. It has been suggested that the long branch chains of amylopectin could bind with iodine in the apparent amylose content methodology, developing a blue color [24]. Thus, the increase in apparent amylose content may be the effect of iodine binding with long amylopectin chains. The results support the statement that the enzymes hydrolyze the amylopectin or the amylose according to the enzymatic treatment [1].

Table 1 shows that both varieties present different amounts of amylose–lipid complexes, with INIAP 650 having high amounts. This result could be due to the higher lipid content reported in INIAP 650 [11]. During hydrolysis, the amylose–lipid complex index in INIAP 650 remained constant, while in INIAP 651 it increased at 30% hydrolysis. The ANOVA analysis reveals that the variety (*p* < 0.001) and hydrolysis processes (*p* < 0.01) influence the amylose lipid complexes index.

Regarding the hydration properties, WBC and WHC significantly increased after hydrolysis in INIAP 651, whereas 50% hydrolysis of INIAP 650 was needed to significantly affect those properties. Both varieties showed a significant increase in WSI. On the other hand, the WAI and the OAC decreased significantly (*p* < 0.05) with hydrolysis in both cassava varieties; thus, hydrolysis increases the hydrophilicity of the starch. The ANOVA analysis shows that the variety (*p* < 0.05) and the hydrolysis process (*p* < 0.01) affect WBC and WAI. Although the ANOVA analysis shows that WHC and WSI were not significantly affected by the variety (*p* > 0.05), these findings could demonstrate that the variety influences the response to enzymatic hydrolysis.

In general, the increase in hydration properties is in good agreement with the expected properties of porous starch in previous studies, which have been related to an increase in surface area during enzymatic hydrolysis [3,4,12,19]. A strong positive correlation between amylose content and WBC (r = 0.75, *p* < 0.01), WHC (r = 0.78, *p* < 0.001) and WSI (r = 0.86, *p* < 0.001) was found. The correlation between amylose and WSI can be explained by the fact that amylose becomes more water-soluble with temperature.

Concerning OAC, different results have been reported. Han et al. [3] reported an improvement in the oil absorption capacity of maize starch when AM, GA, and their mix are used [3]. Their results suggested that the difference in OAC between each treatment depends on the different dimensions of porous channels formed after hydrolysis. On the other hand, no clear tendency of OAC was observed by Benavent-Gil and Rosell [4]. They found a reduction in OAC in cassava starch and an increase of OAC in wheat starch when AMG was used [4]. Furthermore, when AM was used, the OAC of cassava starch was reduced. They attributed this tendency to the difference in starch amylose/amylopectin ratio from different botanical sources. In contrast to these early findings, no significant correlation was found between amylose content and OAC. Table 1 also shows that the OAC results are significantly (*p* < 0.005) influenced by variety and not by the hydrolysis process (*p* > 0.05). In general, as in previous findings, the OAC of both varieties decreased with the hydrolysis, but in INIAP 650 at 30% of hydrolysis the OAC increases. The decrease in OAC could be linked to the strength of the starch granule to support the enzyme attack, which could differ with the botanical source and the variety. In the case of the cassava, mainly exo-hydrolysis occurs, so the starch helix’s lipophilic structure was not exposed.

**Figure 2:**
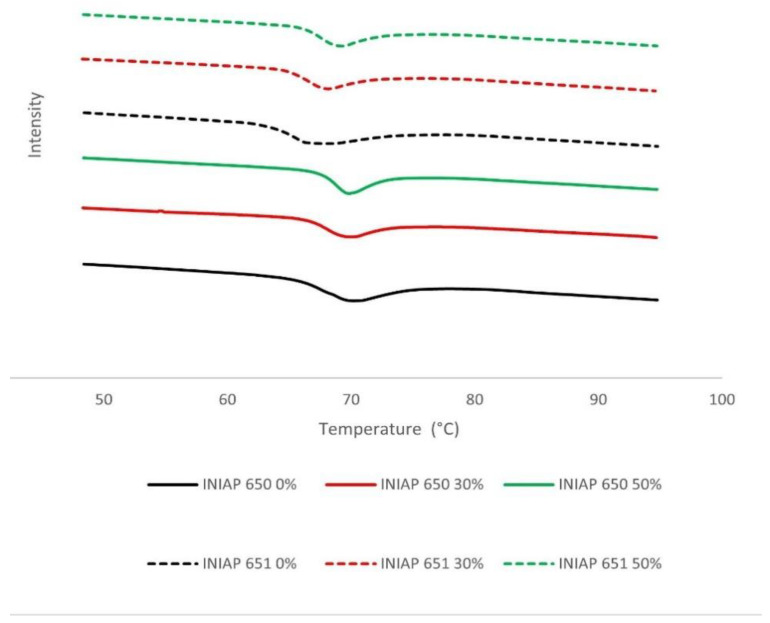
DSC spectra of different cassava starch varieties (INIAP 650, INIAP 651) and hydrolysis degree (0, 30, 50%).

The DSC spectra of different cassava starch varieties (INIAP 650, INIAP 651) and hydrolysis degrees (0, 30, 50%) are shown in Figure 2. The gelatinization temperatures (To, Tp and Tc) were significantly (*p* < 0.05) affected by the variety and starch hydrolysis. The INIAP 650 variety showed a higher gelatinization temperature than the INIAP 651 variety. Unlike what was observed in the hydration properties, the thermal properties between varieties present the same change behavior (Table 1). The onset temperature (To) increases significantly (*p* < 0.05) during hydrolysis in both varieties, and the conclusion temperature (Tc) decreases considerably at 30% of the hydrolysis and after that remains constant at 50%. A different behavior was observed with the peak temperature (Tp); a constant Tp was observed in the INIAP 650 variety and an increase in the INIAP 651 variety.

Diverse findings have been reported on gelatinization temperatures in porous starches obtained by enzymatic hydrolysis. Chen et al. [25] reported a decrease of To, Tp, and Tc in cassava starch when GA and AM were used [25]. On the other hand, Benavent-Gil and Rosell [1] reported no change of To and a decrease in Tp and Tc in cassava starch when AM was used. Recently, an increase in To, Tp, and Tc was reported in corn and rice porous starch [6,12]. Usually, increased gelatinization temperatures are linked to starch crystallinity [6]. However, a strong negative correlation was observed between To with crystallinity (r = −0.79, *p* < 0.005), and no correlation was found with the other gelatinization temperatures.

Some explanations have been proposed for explaining the increase of To. Keeratiburana et al. [16] suggested that this increase could be related to the difficulty of starch granules swelling during gelatinization. Indeed, the correlation analysis showed that To has a negative correlation with WAI (r = −0.90, *p* < 0.001) and a positive correlation with WSI (r = 0.85, *p* < 0.001); thus, these results corroborate those reported by Keeratiburana et al. [16]. In addition, a strong positive correlation was found between amylose–lipid complex with To (r = 0.65, *p* < 0.005) and Tp (r = 0.91, *p* < 0.001). It has been suggested that the amylose–lipid complex restricts the starch granule swelling during gelatinization, increasing the gelatinization temperature [26].

Some authors have suggested that enzymatic hydrolysis co-occurs with the annealing process; it takes place unintentionally [8,16,22]. Annealing is a hydrothermal treatment in which the amorphous region of the starch granule is reorganized, becoming glassier [16]. The increase in gelatinization temperatures has been linked to the annealing process. Furthermore, higher To values have been associated with the relative chain length of amylopectin short-chain [23]. Consequently, the increase of To in both varieties and Tc value in INIAP 651 could be related to two simultaneous processes. The enzymatic hydrolysis increases the amount of amylopectin with shorter branch chains, and the annealing process alters the amorphous region of the starch.

The enthalpy of gelatinization (ΔH) and the gelatinization temperature range (Ig) decreased with the hydrolysis intensity but remained constant at extended hydrolysis (>30% of hydrolysis), displaying the same behavior as Tc. The correlation analysis revealed that these three properties (ΔH, Ig, and Tc) have a strong negative correlation (r < −0.71; *p* < 0.01) with amylose. Furthermore, the correlation analysis indicates that ΔH and Ig presented a negative correlation with WSI (r < −0.85; *p* < 0.001) and with To (r < −0.76; *p* < 0.01) and showed a positive correlation with WAI (r > 0.79; *p* < 0.001). No significant difference was found between varieties in both properties (ΔH and Ig). The decrease in ΔH showed that less energy was necessary for gelatinization. Whereas Chen et al. [25] presented an increase of ΔH in cassava starch, they suggested that this result is attributable to the double helices disruption in the non-crystalline regions of the starch granule during hydrolysis. In contrast, other studies have shown a reduction in ΔH with enzymatic hydrolysis [1,4,6,25]. According to Lacerda et al. [6], the reduction of ΔH was attributed to the loss of double helices and some crystallites during hydrolysis [6]. However, no correlation with crystallinity was found.

**Figure 3:**
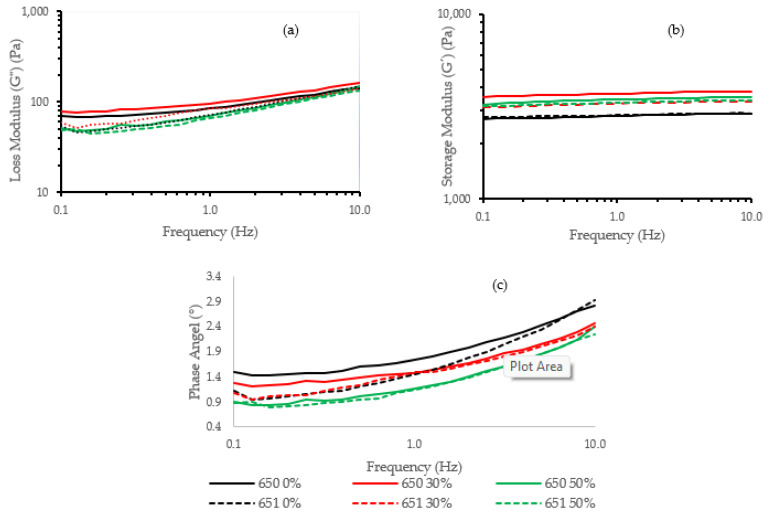
(**a**) Dynamic frequency sweep moduli (*G*′, *G*″) and (**b**) phase angle of different cassava starch varieties (INIAP 650, INIAP 651) and (**c**) hydrolysis degree (0, 30, 50%).

Figure 3 shows the rheological behavior of 650 and 651 cassava starch gels hydrolyzed at 0%, 30%, and 50%. In general, the storage module (*G*′) values (Figure 3b) are higher than the loss module (*G*″) values (Figure 3a), and the phase angle is lower than 45° (Figure 3c), demonstrating that the cassava gels are in a glassy region with a solid-like behavior. In the figure, the loss module (*G*″) values are higher in the INIAP 650 variety than the INIAP 651 variety, showing a more viscous gel. Both varieties presented an increase of *G*″ at 30% hydrolysis. The storage modulus increased in both varieties after hydrolysis, but the extent of the hydrolysis did not further modify their elasticity. The ANOVA analysis indicates that the hydrolysis process influences *G*″ (*p* < 0.01) (Table 1). At 1 Hz, no significant difference was observed in the storage module (*G*′) between varieties or hydrolysis degrees (Table 1). For the phase angle, a decrease was observed with the hydrolysis, being more significant in the INIAP 650 variety than the INIAP 651 variety (Figure 3c). The reduction of the phase angle shows that the gel becomes stronger with hydrolysis.

### 3.3. Principal Component Analysis

**Figure 4:**
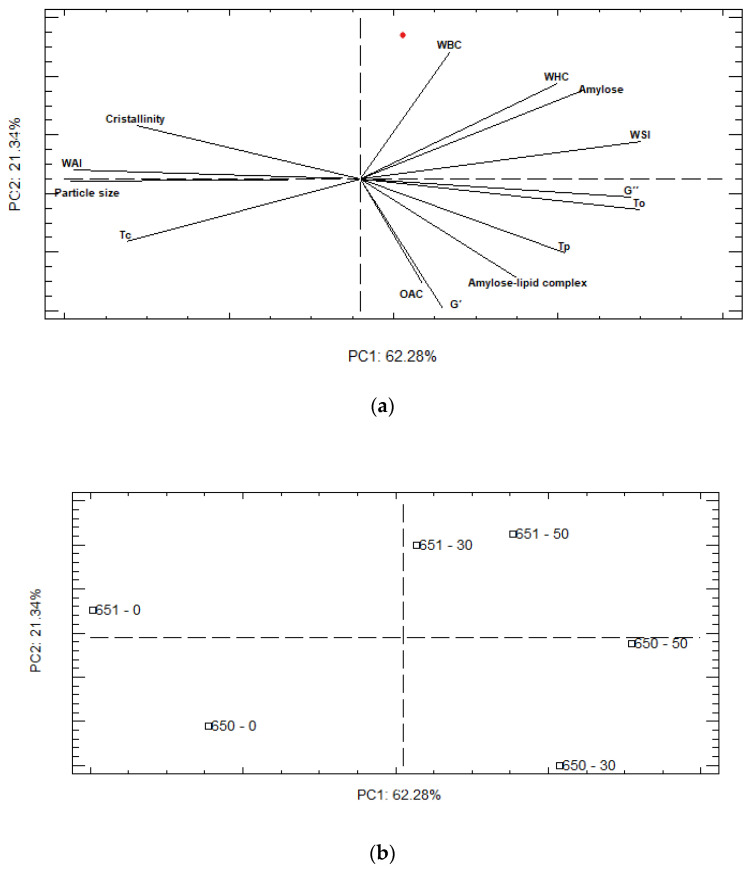
Analysis of principal components (**a**) loading and (**b**) score plots. WBC: water binding capacity, WHC: water holding capacity, WAI: water absorption index, WSI: water solubility index, OAC: oil absorption capacity. To: onset temperature, Tp: peak temperature, Tc: conclusion temperature.

A principal component analysis (PCA) was performed to construct a global picture of porous starch characteristics, using structural, thermal, functional, and rheological properties (Figure 4). PC-1 (62.28%) and PC-2 (21.34%) explained 83.62% of the total variation in these parameters. The representation of the scores plot (Figure 4b) confirms a stronger effect of the hydrolysis process over both varieties, with 0% of hydrolysis appearing in the opposite corner of PC1, from 30 and 50% hydrolysis. In addition, the PCA analysis revealed that the INIAP 650 and INIAP 651 varieties respond differently to the hydrolysis process. As shown in Figure 4, in the INIAP 650 variety, 30% and 50% of hydrolysis appears in the negative axis of PC-2, and they are characterized by higher viscoelastic parameters, To, Tp, and also OAC. In the INIAP 651 variety, 30% and 50% of hydrolysis are segregated along positive PC-2, which were related to higher hydration parameters. This evidence shows that the parameters of the INIAP 651 variety remain constant after 30% of hydrolysis, while in the INIAP 650 variety, the parameters change.

## 4. Conclusions

The evidence from this study suggests that the response to the hydrolysis process of cassava starch from two varieties is significantly different. The INIAP 651 variety presents more severe damage in its structure due to hydrolysis, significantly affecting its physicochemical properties. The results obtained in the thermal properties corroborate previous studies in that there is also an “annealinG″ or cooking process during the partial enzymatic hydrolysis of the starch. The present study reveals that the variety affects the starch granule’s response to enzymatic hydrolysis.

## Figures and Tables

**Table 1:** Structural and physicochemical properties of different cassava starch varieties and hydrolysis grade.

Variety	INIAP 650		INIAP 651			*p* Value *		
% Hydrolysis Degree	0%	30%	50%	0%	30%	50%	Variety	Hydrolysis
Crystallinity (%)	25.70 ± 1.90 ^b,c^	21.50 ± 1.03 ^a,b^	18.98 ± 0.39 ^a^	27.06± 2.64 ^c^	23.72 ± 3.39 ^a,b,c^	28.22 ± 1.57 ^c^	0.022	0.025
Particle < size D (4, 3) (µm)	6.65 ± 1.44 ^b,c^	5.83 ± 1.38 ^a,b^	5.81 ± 1.03 ^a,b^	7.06 ± 1.70 ^c^	6.05 ± 1.19 ^a,b^	5.96 ± 1.12 ^a,b^	0.096	0.014
Amylose (g/100 g)	23.68 ± 0.33 ^a^	26.24 ± 0.51 ^b^	28.57 ± 0.77 ^c^	24.46 ± 0.19 ^a^	28.24 ± 0.38 ^c^	30.46 ± 0.19 ^d^	<0.001	<0.001
Amylose–lipid complex	26.52 ± 1.09 ^c^	26.45 ± 1.11 ^c^	27.54 ± 0.83 ^c^	7.34 ± 0.82 ^a^	14.96 ± 0.62 ^b^	14.19 ± 1.60 ^b^	<0.001	0.001
WBC (g/g)	1.01 ± 0.07 ^a^	0.99 ± 0.07 ^a^	1.16 ± 0.04 ^b,c^	1.09± 0.04 ^a,b^	1.18 ± 0.01 ^c^	1.20 ± 0.08 ^c^	0.010	0.008
WHC (g/g)	0.86 ± 0.03 ^a^	0.87 ± 0.01 ^a^	1.00 ± 0.06 ^b,c^	0.87 ± 0.03 ^a^	0.95 ± 0.07 ^b^	1.02 ± 0.05 ^b,c^	0.081	<0.001
WAI (g/g)	12.37 ± 0.12 ^c^	6.22 ± 0.29 ^a^	6.78 ± 0.34 ^a^	12.91 ± 0.43 ^d^	8.47 ± 0.15 ^b^	8.57 ± 0.24 ^b^	<0.001	<0.001
WSI (g/100 g)	0.05 ± 0.00 ^a^	3.52 ± 0.64 ^b^	4.53 ± 0.23 ^c^	0.05 ± 0.01 ^a^	3.80 ± 0.20 ^b^	3.73 ± 0.31 ^b^	0.329	<0.001
OAC (g/g)	1.99 ± 0.06 ^b^	2.88 ± 0.01 ^c^	1.89 ± 0.07 ^a^	2.05 ± 0.10 ^b^	1.81 ± 0.04 ^a^	1.80 ± 0.01 ^a^	0.031	0.434
To (°C)	65.04 ± 0.35 ^b^	66.96 ± 0.15 ^c^	67.81 ± 0.09 ^d^	62.99 ± 0.30 ^a^	65.31 ± 0.07 ^b^	66.42 ± 0.03 ^c^	<0.001	<0.001
Tp (°C)	69.40 ± 0.14 ^d^	69.51 ± 0.15 ^d^	69.86 ± 0.10 ^d^	67.29 ± 0.27 ^a^	67.79 ± 0.26 ^b^	68.90 ± 0.02 ^c^	<0.001	<0.001
Tc (°C)	81.28 ± 0.82 ^d^	74.01 ± 0.59 ^a,b^	74.07 ± 0.09 ^a,b^	79.57 ± 1.80 ^c^	72.33 ± 0.48 ^a^	73.01 ± 0.06 ^a^	0.002	<0.001
∆H (J/g)	3.28 ± 0.30 ^b^	1.74 ± 0.07 ^a^	1.66 ± 0.08 ^a^	3.32 ± 0.26 ^b^	1.60 ± 0.14 ^a^	1.38 ± 0.12 ^a^	0.142	<0.001
Ig (°C)	16.20 ± 0.52 ^b^	7.04 ± 0.74 ^a^	6.26 ± 0.00 ^a^	16.59 ± 1.92 ^b^	7.02 ± 0.55 ^a^	6.59 ± 0.09 ^a^	0.636	<0.001
*G*′ (Pa)	85.28 ± 2.81 ^a^	95.83 ± 3.33 ^a^	85.18 ± 32.27 ^a^	72.28 ± 20.15 ^a^	70.48 ±16.23 ^a^	65.76 ± 2.05 ^a^	0.253	0.289
*G*″ (Pa)	2804.50 ± 153.44 ^a^	3706.00 ± 171.12 ^b^	3441.00 ± 381.84 ^b^	2832.00 ± 275.70 ^a^	3278.50 ± 7.79 ^a,b^	3312.50 ± 74.25 ^a,b^	0.235	0.005

The results were presented as mean ± standard deviation. Values followed by different letters (a, b, c, d) within a row denote significant differences (*p* < 0.05) in the parametric and non-parametric data analysis. WBC: water binding capacity, WHC: water holding capacity, WAI: water absorption index, WSI: water solubility index, OAC: oil absorption capacity. To: onset temperature, Tp: peak temperature, Tc: conclusion temperature, ΔH: enthalpy change, Ig: gelatinization interval. * Values *p* for analysis variance of physicochemical properties of hydrolyzed starch.

## Data Availability

The data analyzed in this study are available from the authors upon reasonable request.
